# Who remains at the margins of women’s health? Sexual and gender diversity in research and care

**DOI:** 10.4069/whn.2026.09.17

**Published:** 2026-09-29

**Authors:** Sook Jung Kang

**Affiliations:** College of Nursing, Ewha Womans University, Seoul, Korea

Women’s health has expanded beyond its primary focus on reproductive health to encompass health across the lifespan. This broader perspective recognizes how biological factors, gender, socioeconomic position, access to health care, and public policies shape health [1]. However, important gaps in evidence and care remain for some populations. For transgender and gender-diverse people, sampling and measurement challenges limit research on sexual and reproductive health, while barriers to appropriate care persist [2]. Gender-diverse populations may remain among those at the margins of women’s health. In this editorial, lesbian, gay, bisexual, transgender, and queer or questioning people and other sexual and gender minority populations (LGBTQ+) are considered collectively. Their health needs vary, and some extend beyond the scope of women’s health. LGBTQ+ may nevertheless need services that overlap with women’s health practice, including sexual, reproductive, gynecological, preventive, and mental health care. The aim of this editorial is to clarify who is included, who is overlooked, and where avoidable gaps remain, while retaining women as a population of concern.

## Evidence gaps and inequities in care

The limited evidence from Korea nevertheless points to important gaps. A 2024 narrative review identified only 33 quantitative studies on the health of Korean LGBTQ+ populations published through February 2024; most focused on mental health and health-related behaviors [3]. In a national study of transgender and gender-diverse individuals, 68.4% reported having avoided or postponed health care. Repeated discrimination related to gender identity or expression was associated with a 78% greater likelihood of avoiding or delaying non-transition-related health care, possibly because of previous mistreatment, disrespect, or insufficient knowledge among providers, and anticipated rejection or stigma [4]. Earlier Korean research likewise found that transgender adults who had experienced discrimination were more likely to avoid seeking health care [5].

Together, these findings highlight a persistent problem in women’s health: populations with substantial health needs may remain underrepresented in research and underserved by health care. Health equity cannot be assessed if research is based on data from an unrepresentative population. Assessing equity requires attention to those who are least visible, least represented, or least able to benefit from advances in care. Although research and professional discourse increasingly recognize gender diversity, this recognition has not consistently informed research and practice. The increasing use of digital health care may intensify these concerns. Digital technologies may improve access and support more individualized care, but inequities in access, data representation, and engagement may perpetuate or even widen existing disparities [6,7]. Incomplete or unrepresentative data may have particularly important consequences for sexual and reproductive health because sensitive information, privacy, and unequal access are already central concerns. These issues are central to women’s health research, which has long examined the intersections of biological processes with gendered roles, caregiving, social conditions, and unequal access to resources. Addressing sex-specific differences is compatible with recognizing gender diversity. When relevant, sex should be measured accurately, and gender identity should be treated as a distinct variable where appropriate [8].

## The need for institutional commitment

International experience suggests that established institutional structures are more likely to sustain progress in LGBTQ+ health than the efforts of individual researchers alone. At Columbia University School of Nursing, the Center for Sexual and Gender Minority Health Research combines research, investigator development, and mentorship with attention to the social determinants of health [9]. Similarly, the University of Pennsylvania School of Nursing’s Eidos LGBTQ+ Health Initiative combines research and education with community partnership, evaluation, and social innovation [10]. Nationally, the United States National Institutes of Health (NIH) has maintained an NIH-wide strategy for sexual and gender minority health research and is developing its 2026–2030 strategic plan [11].

Although these initiatives differ in scope and function, they share sustained institutional commitment, dedicated funding, training, collaboration, and clear pathways from evidence to practice. The nursing profession has also begun to articulate its responsibilities in this area. The National Nursing LGBTQ Health Summit called for more systematic integration of sexual and gender minority health into nursing curricula, clinical guidelines, faculty and continuing education, and research priorities [12]. Midwifery education provides an example of such a commitment in practice: a whole-of-program approach integrated LGBTQ+ health equity into courses, assessments, clinical learning, and informal teaching rather than confining it to a single session [13].

## Priorities for women’s health nursing in Korea

These considerations point to three priorities. First, women’s health research needs to make underrepresented populations more visible. When relevant to the research question, sex, sexual orientation, and gender identity should be assessed separately and reported in enough detail to support meaningful interpretation. This does not require every study to collect every variable. Researchers should instead limit data collection to the information needed to answer the research question, protecting privacy and avoiding unnecessary collection of sensitive data. More precise measurement can preserve the importance of sex-based evidence while revealing differences obscured by broad categories.

Second, Korea needs more sustainable research infrastructure. Progress is difficult to maintain when expertise rests with a small number of researchers and depends largely on short-term project support. Conducting research in Korea presents further challenges that compound these limitations. Stigma and the risk of unwanted disclosure may deter participation, making efforts to build trust and protect confidentiality particularly important in Korea [14]. For example, a mismatch between the sex-designating digit in a Korean resident registration number and a transgender individual’s gender identity may involuntarily reveal that person’s transgender status [14]. Targeted pilot funding, support for early-career researchers, interdisciplinary networks, and university-based research hubs could provide the continuity needed to accumulate expertise, strengthen data resources, and sustain community partnerships. The initiatives at Columbia University and the University of Pennsylvania illustrate how schools of nursing can support this infrastructure [9,10].

Third, researchers should examine whether treatments and interventions are equally applicable and effective across sexes and gender identities, rather than assume that their effects are uniform. Throughout their curricula, nursing schools should ensure that students acquire a foundational understanding of LGBTQ+ populations and their health care needs. Textbooks across nursing specialties, including women’s health nursing, should explain how specific conditions may present and how assessments and nursing interventions may need to be adapted for LGBTQ+ individuals. These topics should also be incorporated into clinical training and continuing education programs, enabling nurses at all career stages to update their knowledge and apply it in practice.

These priorities must remain a focus of research design, research conduct, and care delivery. As health care becomes increasingly data-driven and digitally mediated, nursing must remain vigilant to ensure that existing exclusions are neither reproduced nor worsened. Women’s health nursing is especially well positioned to lead this work.
